# The pathogenicity of *Plasmopara viticola*: a review of evolutionary dynamics, infection strategies and effector molecules

**DOI:** 10.1186/s12870-024-05037-0

**Published:** 2024-04-24

**Authors:** Catarina Gouveia, Rita B. Santos, Catarina Paiva-Silva, Günther Buchholz, Rui Malhó, Andreia Figueiredo

**Affiliations:** 1 Biosystems and Integrative Sciences Institute (BioISI), Faculty of Science, University of Lisbon, Lisboa, Portugal; 2 RLP AgroScience/AlPlanta-Institute for Plant Research, Neustadt an Der Weinstrasse, Germany

**Keywords:** Oomycetes, Host–pathogen interaction, Downy mildew, Grapevine, Plant defence, Pathogen effectors

## Abstract

Oomycetes are filamentous organisms that resemble fungi in terms of morphology and life cycle, primarily due to convergent evolution. The success of pathogenic oomycetes lies in their ability to adapt and overcome host resistance, occasionally transitioning to new hosts. During plant infection, these organisms secrete effector proteins and other compounds during plant infection, as a molecular arsenal that contributes to their pathogenic success. Genomic sequencing, transcriptomic analysis, and proteomic studies have revealed highly diverse effector repertoires among different oomycete pathogens, highlighting their adaptability and evolution potential.

The obligate biotrophic oomycete *Plasmopara viticola* affects grapevine plants (*Vitis vinifera* L.) causing the downy mildew disease, with significant economic impact. This disease is devastating in Europe, leading to substantial production losses. Even though *Plasmopara viticola* is a well-known pathogen, to date there are scarce reviews summarising pathogenicity, virulence, the genetics and molecular mechanisms of interaction with grapevine.

This review aims to explore the current knowledge of the infection strategy, lifecycle, effector molecules, and pathogenicity of *Plasmopara viticola*. The recent sequencing of the *Plasmopara viticola* genome has provided new insights into understanding the infection strategies employed by this pathogen. Additionally, we will highlight the contributions of omics technologies in unravelling the ongoing evolution of this oomycete, including the first in-plant proteome analysis of the pathogen.

**Supplementary Information:**

The online version contains supplementary material available at 10.1186/s12870-024-05037-0.

## Background

Oomycetes are a group of organisms with filamentous growth that resembles the morphology and life cycles of fungi (Eumycota), mainly due to convergent evolution. Like phytopathogenic fungi, oomycetes can absorb nutrients directly from the host, sharing some convergent traits with fungi, such as filamentous growth in the vegetative stage, mycelia and spores for asexual and sexual reproduction [1]. Despite these common features, there are many differences that distinguish oomycetes, such as the presence of biflagellate motile stages with two different types of flagella, whiplash or tinsel. Tinsel flagella are typical of brown algae and diatoms, contributing to the phylogenetic proximity between Oomycota and Straminipila [2]. Ploidy level is also different throughout the life cycles of oomycetes and fungi. While oomycete vegetative stages are either diploid or polyploid, most fungi are haploid or dikaryotic across their life cycle. Additionally, oomycetes present a cellulose-based cell wall, whereas fungi have chitin-based cell wall [3].

Oomycetes are thought to have branched from the diatom group around 0.4 to 0.6 billion years ago, with phytopathogenicity evolving independently in several lineages [4]. Oomycetes can be divided into several orders, and their phylogeny has been extensively explored in two recent studies [4, 5]. The four most relevant orders are Saprolegniales, Peronosporales, Albuginales, and Pythiales. The order Saprolegniales mainly includes fish pathogens and phytopathogens from marine and freshwater ecosystems, whereas most of the oomycetes in the Albuginales, Pythiales, and Peronosporales orders are phytopathogens. The order Peronosporales includes pathogens from terrestrial ecosystems, such as the genus *Phytophthora, Plasmopara* and *Hyaloperonospora* [4].

The evolutionary success of pathogenic oomycetes is rooted in their ability to adapt to and overcome host resistance, occasionally transitioning to new hosts. The flexible mating system of oomycetes (sexual or asexual reproduction or interspecific hybridization) provides an evolutionary advantage to these organisms [5]. While not limited to oomycetes, their ability to release a range of molecular weapons, including effector proteins, during plant infections significantly contributes to their success. Genome sequencing of different oomycete species, as well as transcriptomic and proteomic studies, have compared effector repertoires between different pathogens and showed that they are highly diverse [5].

The European grapevine (*Vitis vinifera* L.) is one of the most cultivated fruit plants worldwide and has a very high economic impact [6]. However, it is susceptible to diseases introduced into the wine-growing regions by human activities such as downy mildew. This disease is caused by the obligate biotrophic oomycete *Plasmopara viticola*. Although some reviews have been published focusing on pathogen history, epidemiology, infection, and control mechanisms [3, 7], several advances into our understanding of the pathogen’s infection strategy were made after *Plasmopara viticola* genome release [8–10]. Given the importance of this pathogen and its impact in viticulture, in this review we will summarise the current knowledge on its pathogenicity mechanisms and effector molecules. We will also discuss how Omics-based research can assist the study of the evolution of this pathogen, highlighting the main conclusions drawn from the first in-plant proteome of *P. viticola.*

### The grapevine downy mildew pathogen: *Plasmopara viticola*

*Plasmopara viticola* (Berk. & Curtis) Berl & De Toni, the etiological agent of grapevine downy mildew, belongs to the oomycete family Peronosporaceae. As an obligate biotrophic pathogen, *P. viticola* grows only on living grapevine tissues and is considered one of the main causes of production losses of several million euros. In temperate and humid regions, downy mildew can attack all green plant organs, such as leaves, tendrils, shoots, and clusters [7]. The development of the disease is favoured by mild temperatures and high humidity (e.g., spring rain).

**Fig. 1:**
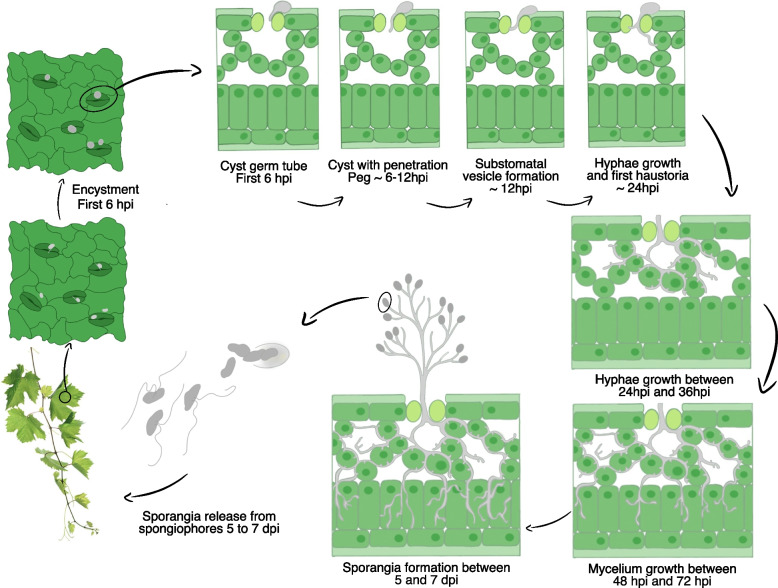
Scheme of *Plasmopara viticola* asexual life cycle

Oospores, the overwintering form in the *P. viticola* life cycle, can take up to 7–10 days to germinate depending on adequate climatic conditions. When these conditions are met, they germinate, releasing zoospores which move in the water film and encyst at the stomata on the lower surface of leaves or other green tissues (Fig. 1). After encysting, the zoospores germinate and develop a germ tube that penetrates the stomata into the substomatal cavity (primary hyphae) and the parenchyma. Then, the primary hyphae branch into the intercellular space of the mesophyll and form haustoria, which remain in contact with the plasma membrane. Haustoria spread throughout the leaf parenchyma, allowing the pathogen to obtain nutrients, suppress defence mechanisms, and redirect the host's metabolism to its favour [11]. Consequently, the quintessential symptom of grapevine downy mildew appears in susceptible hosts, characterized by yellowish spots termed oil spots. After the appearance of the oil spots as the first symptoms, under favourable conditions (relative humidity > 90%, temperatures between 20–25 ºC), sporangiophores emerge from the stomata carrying sporangia, enabling secondary infections (Fig. 1). Several cycles of clonal infections can occur during the vegetation period, seriously compromising the quality and quantity of grape production. Late infections in the vegetation period led to the formation of oospores after conjugation of the sexual gametangia (oogonia and antheridia) [12], which can hibernate in leaf litter and soil until the following season [13].

### Host speciation

With the advances in *P. viticola* genomic information, initial steps into the understanding of pathogenicity and virulence are being given. In fact, genome organisation and the identification of chromosome regions that are more prone to selective pressure have pointed out to a co-evolutionary dynamic between *P. viticola* and grapevine [14]. Host shifts may contribute to these dynamics. In fact, the intimate relation between hosts and pathogens lead to the evolution of host-specific adaptations [15]. In *P. viticola* center of origin (North America) this pathogen interacts with several Vitis species, and the existence of race-specific isolates was pointed out [16]. In 2013, Rouxel presented genetic, morphological, and virulence-based evidence to the existence of a complex of cryptic species that have radiated in Vitaceae and that presented host plant specialization [15].

Up to date, in the *P. viticola* species complex, five cryptic species were identified presenting genetic differentiation and different host ranges [15, 17]. These five *P. viticola formae speciales* (f. sp.) are: (1) *P. muralis*, with a host plant specialisation towards *Parthenocissus quinquefolia* and *V. riparia;* (2) *P. viticol*a clade *riparia*, that is only able to infect interspecific hybrids; (3) *P. viticola* clade *vinifera*, that is able to infect *V. vinifera* cultivars and hybrids; (4) *P. viticola* clade *vulpina*, found in *V. vulpina* and (5) *P. viticola* clade *aestivalis* which is the main clade present in *V. vinifera* vineyards [15, 17]. Furthermore, the clade f. sp. *aestivalis* was pointed out as responsible for the initial colonisation of European vineyards, ultimately serving as the source for the introduction of *P. viticola* to vineyards across the world [18].

Regarding *P. viticola* clades, high genetic diversity was also reported [15, 17, 19]. In fact, *P. viticola* clades present frequent sexual recombination leading to high recombination rates. In this sense, one may hypothesise that different species may be hidden within these “clades”. This was previously pointed out by Schröder et al. that studied several isolates from vineyards in the United States with both nuclear and mitochondrial markers, leading to the identification of three lineages that present significant genetic divergence [19].

### *Plasmopara viticola* genetic diversity

While North America is considered the centre of *P. viticola* origin, European *Vitis vinifera* only had contact with *P. viticola* in 1878, when this pathogen was introduced in the European continent [7]. After its introduction, the grapevine downy mildew disease became epidemic leading to severe yield losses. Only in 1998 the scientific community began to characterise *P. viticola* population genetic structure in Europe. Random amplified polymorphic DNA (RAPD) markers were then used to estimate the pathogen genetic diversity in Switzerland vineyards [20]. Since then, several attempts have been conducted to explore the genetics of this pathogen population with two types of approaches being mainly used in European vineyards throughout the years: RAPD markers [21] and the microsatellites/simple sequence repeats (SSR markers) [22–28].

Recent findings regarding the global dispersion patterns of *P. viticola* pointed out to Europe as a source for *P. viticola* introduction worldwide [18]. In this study, Fontaine et al. 2018 used mitochondrial gene sequences and microsatellite markers to understand the pathogen introduction and dispersal routes and by studying 2000 isolates from the main world wine producing regions (North America—native pathogen; Europe, China, South Africa, Australia, and Argentina) drawn several conclusions: cultivated European vineyards were devastated by a specific genetic cluster of *P. viticola sp. aestivalis* and European populations of this pathogen were the sources for secondary introduction in other continents, such as China, South Africa and Australia [18, 29–32]. For South Africa the analysis of *P. viticola* population throughout different seasons suggested that population structure in warmer climates (such as South Africa and Australia) might be different than what is found in regions with more favourable environmental conditions [31]. These populations would suffer bottlenecks during unfavourable climatic conditions leading to a strong selective pressure.

Altogether these approaches pointed out to some major conclusions, namely: (1) genetic diversity is higher in North America populations (founder effect); (2) *P. viticola* populations in the regions where it was first introduced have high genetic diversity relative to other European populations; (3) most of the genetic diversity present in European *P. viticola* is found within small spatial scales (e.g.: vineyards), however despite no data on long-distance dispersal is yet available, we should not ignore its importance; (4) two genetic clusters of *P. viticola* are present in Europe (Eastern and Western Europe), which may point out to distinct waves of pathogen expansion; (5) primary infections are important for the epidemics and oosporic infections are important during the growing season; (6) continental-wide population structure in Europe is weak (although significant) and (7) *P. viticola* populations act as panmictic units with prevalent sexual recombination happening every winter.

### *Plasmopara viticola* adaptation: the overcome of grapevine resistance

The publication of *P. viticola* high quality genome assembly [8] enabled the study of mechanisms involved in the adaptation to biotic and abiotic selective pressures, allowing a better understanding of population genomics. Through this, it was shown that several genes encoding for secreted proteins and RxLRs present a fast evolution rate and are involved in pathogen adaptation [8] to biotic and abiotic factors. This feature is highlighted in several studies that pinpoint the breakdown of grapevine resistance [14, 33–36]. Understanding the breakdown of plant resistance is of utmost importance when aiming for a more sustainable viticulture, especially following the pesticide use restrictions imposed by the European Union.

*Plasmopara viticola* and American grapevine species have evolved together, so American genotypes are genetically resistant to this pathogen. Thus, American germplasm has been widely used in grapevine breeding programs as a source of resistance traits. Plants obtained through breeding programs using hybrids of *V. vinifera* and resistant/tolerant *Vitis* species exhibit greater tolerance to the disease, all while maintaining the desirable characteristics of *V. vinifera*, constituting a viable alternative to the use of pesticides. In this field, scientific research has contributed to the identification of regions in grapevine chromosomes where resistance genes to this pathogen are located. Currently, 31 resistance loci associated with *P. viticola* resistance (Rpvs) have been described [37], 27 of which are listed in the database of the International Variety Catalogue of Grapevine (Vitis International Variety Catalogue-VIVC, www.vivc.de]). There are several examples of commercially available varieties that were bred for resistance such as ‘Regent’ (Rpv3.1; VIVC number 4572), ‘Solaris’ (Rpv10, Rpv3.3; VIVC number 20340) and ‘Bianca’ (Rpv3, Rpv7 and Rpv11; VIVC number 1321).

One of the most widely harnessed resistance locus in breeding programs is the Rpv3 locus [38], followed by Rpv10 and Rpv12. However, and perhaps because of this, the emergence of *P. viticola* isolates capable of breaking down plant resistance has been shown for these three backgrounds [35, 39, 40]. Recently, Paineau et al. 2022 analysed 33 European *P. viticola* pathovars virulence towards different host resistance factors. Five pathotypes were shown to be able to overcome either single or multiple resistance factors [40]. In fact, of the 33 pathovars analysed, 28 were able to overcome Rpv3 haplotypes confirming the widespread occurrence of host resistance breaking isolates.

In fact, while host resistance genes have been widely studied, knowledge on *Plasmopara viticola* avirulence factors is scarce. Due to the establishment of host hypersensitive reaction, it is hypothesised that a gene-for-gene interaction may be occurring between grapevine and *P. viticola*. Nonetheless, no AVR-R gene partners were identified so far. Several attempts are being conducted to identify avirulence factors in *Plasmopara viticola*, namely the recent full genome sequencing of 136 *P. viticola* strains sampled in a natural population of Bordeaux leading to the development of genome-wide association studies enabled the identification of a Rpv3.1 breakdown related region in *P. viticola* [14]. This approach led to the identification of the avrRpv3.1 locus involved in the interaction with the host Rpv3.1 loci, which, based on population genetics indices, was pointed out to be under positive selection on resistance hosts. This locus presents major structural variations together with a 30Kbp deletion, which may represent an evolutionary strategy, as deletion of avirulence genes may aid to evade host recognition [14]. Moreover, a high density of transposable elements was described for this region, which has been shown in another pathosystem to significantly impact the virulence of the pathogen [41].

Another driving force for evolution worth exploring in *P. viticola* lies in its sexual reproduction cycle. Although highly challenging due to the obligatory biotrophic lifestyle of *P. viticola*, Dvorak et al. were able to cross two parental strains of interest, INRAE-Pv1419 (overcomes *Rpv3 and Rpv10*-mediated resistance) and INRAE-Pv412 (overcomes *Rpv3*-mediated resistance). The progeny was evaluated by phenotyping pathogenicity related traits with the aim of understanding the mode of inheritance of the resistance breakdown associated traits [42].

### RXLRs and CNR as weapons in oomycete infection strategy

Phytopathogenic oomycetes rely on a diverse array of enzymes and effector molecules to carry out the intricate infection process. Upon encountering the stomata of the host tissue, the zoospores attach to their guard cells and initiate the infection. Subsequently, the developing infection structures must navigate through, suppress, and manipulate the host’s constitutive and induced defence mechanisms. Most oomycetes employ various tactics, such as secreting adhesins, cell wall-degrading enzymes, elicitors, or effectors, as part of their infection strategy. These effectors, acting as virulence factors or toxins, facilitate the establishment of disease, while others, known as elicitors and/or avirulence factors (Avr), are recognized by the plant, triggering a defence response from the host. Effectors may be recognized by plant receptors on the plant cell surface or can be translocated into the plant cytoplasm after haustoria development [43–45]. Overall, oomycete effectors possess three main characteristics: 1) the ability to trigger or suppress cell death; 2) the translocation to and within the host plant; and 3) the capacity to enhance colonisation of host cells or boost host resistance to the pathogen. These effectors are secreted across the haustorial host–pathogen interface to modulate host cellular processes and enhance disease susceptibility. Among the oomycete effectors, the most well-known classes are RxLR and Crinkler (CRN for CRinkling and Necrosis symptoms on leaves; [34]).

Typically, the RxLR proteins consist of a signal peptide at the N-terminus, followed by an RxLR motif, or its variant, and it is often associated with an EER motif that facilitates secretion and translocation to the plant cell [46]. Also, in many cases one or more WY-domains are present leading to a structural fold specific to these effectors. Moreover, many RxLR proteins present the LWY-domain, often organised in tandem repeats leading to a structural and functional modularity of these effectors and contributing to their high evolutive potential [47].

RxLR proteins are recognized by plant resistance (R) genes, and highly expressed in early time-points of infection [48]. RxLR effectors are present in most oomycete genomes and have been described to overcome R genes because of their high mutation rates and evolutionary potential. For instance, the *Phytophthora infestans* AVR3a effector can evade recognition by the *Nicotiana benthamiana* NLR protein R3a through mutation of two amino acids [49]. Despite their constant evolution, the conserved sequence in some RxLR effectors can help identify their target R genes across different pathogen strains [50, 51].

Although the function of many RXLR effectors is still unknown, increasing research is contributing to the knowledge of their importance and probable function. PvRxLR131 is one of the recently studied effectors that was proven to enhance pathogenicity when overexpressed in *Colletotrichum gloeosporioides* and to decrease the resistance of *Arabidopsis thaliana* to *P. syringae* and of *N. benthamiana* to *P. capsici* when expressed *in planta* [52]. Protein–protein interaction assays have shown that PvRxLR131 effector activity is mediated by BRI1 kinase inhibitor 1 (VvBKI1) to suppress growth- and defense-related brassinosteroid (BR) and ERECTA (ER) signaling [52]. PvRxLR28 was shown to inhibit the expression of defense-related genes, such as ROS-producing proteins. It has been described to enhance susceptibility to *P. viticola* and *P. parasitica* when transiently expressed in the leaves of grapevines and *N. benthamiana*, respectively [53]. In contrast, PvRxLR16 induced the upregulation of defense-related genes, such as those involved in the salicylic acid (SA), jasmonic acid (JA), and ethylene (ET) signalling pathways. Its overexpression in *N. benthamiana* leaves was found to trigger cell death and ROS accumulation and consequently increase disease resistance, thus suggesting that PvRxLR effectors may suppress immune responses triggered by PvRxLR16 [53, 54]. More recently, PvAvh77, another avirulence homolog, has been shown to trigger cell death in *Vitis riparia* and *N. benthamiana*. However, when expressed in *V. vinifera* ‘Thompson seedless’ plants engineered to be tolerant to *Botrytis cinerea* and *Erysiphe necator*, it increased the susceptibility to *P. viticola* by reducing host immunity [55]. Two other *P. viticola* effectors, PvRxLR53 and PvRxLR159, inhibited the induction of programmed cell death by INF1 and BAX when overexpressed in *N. benthamiana*. It was observed that PvRxLR53 suppressed ROS production induced by *P. capsici* and, consequently, reduced plant resistance to this pathogen. PvRxLR159 has also been shown to reduce *N. benthamiana* resistance to *P. capsici* [56]. The expression of these effectors at the initial hours post-inoculation of grapevine leaves suggests that *P. viticola* might secrete PvRxLR53 and PvRxLR159 to suppress host immunity early in the infection process [56, 57].

CRN effectors are a class of small secreted proteins that was first identified in *P. infestans*, which are able to suppress plant defences [58, 59]. This class is characterised by a conversed LxLFLAK motif in the N-terminal region, while the C-terminal domains are highly variable [59]. The CRN effectors in *P. viticola* have been studied by Xiang et al. [59]. These authors identified 27 CRN-like genes in the YL isolate, 15 of which were found to suppress plant cell death induced by BAX. However, only one CRN effector suppressed cell death induced by INF1. PvCRN17, PvCRN20, and PvCRN23 significantly suppressed plant cell death and promoted *P. capsici* pathogenicity in *N. benthamiana*. Overexpression of PvCRN10 and PvCRN26 enhanced the resistance of *N. benthamiana* leaves to *P. capsici*, despite being described as cell death suppressors, indicating an ability to reduce this defense mechanism. Moreover, PvCRN19, an effector that does not trigger or suppress cell death, was found to enhance *P. capsici* pathogenicity [59].

### RXLRs and CNR diversity in *P. viticola*

Since the genome draft release of *P. viticola*, new insights into pathogenicity and infection strategies have been provided, namely through the identification of effectors namely RxLR and CRN (Supplementary Table 1) in different isolates [60, 61]. Prior to genome release, a secretome analysis by de novo transcriptome assembly of three isolates (CRIRO-L-2 from Adelaide, Australia and ZJ-1-1 and JL-7-2 from the NorthEast of China) already pointed out to the existence of 51 combined RxLR effectors [61]. Also, a transcriptomic resource containing *P. viticola* (SL, Czech isolate and SC, French isolate [34] and *P. halstedii* pointed to a common existence of 50 putative RxLR and 60 CNR [62].

After the first genome draft, several works pointed to different numbers of RXLR and CNR genes in the *P. viticola* genome. For the *P. viticola* isolate, INRA-PV221 collected in Bordeaux, France in 2016 [9], 540 RxLR coding genes were identified [8]. Shortly after, the whole genome sequence of JL-7-2 *P. viticola* isolate from Shangai, China, was published and 100 RxLR and 90 CRN coding sequences were predicted. Also in this work, the authors compared the RxLR and CRN gene coding sequences from the JL-7-2 with the already published information on the European isolates (SL and SC; [34]) and found that 18 RxLR and four CRN were common [63]. Later in 2018, Brilli et al., pointed to the existence of 57 RxLR and 68 CRN coding sequences in the PVitFEM01 isolate from San Michele all’Adige, Italy [64]. More recently, the YL isolate, from Shanghai, China, was sequenced and 100 RxLR [65] and 35 CRN were identified. The authors compared the number of RxLR and CNR with the already described for other isolates and compared with JL-7-2 only four genes were found to be common. Compared to INRA-PV221, eight genes were common and finally, when compared to PVitFEM01, only five genes were found to be common [59].

In the high-quality *P. viticola* genome assembly published in 2019, it was pointed out that this pathogen presents a two-speed genome architecture, with secreted protein-encoded genes being located at gene-sparse and repeat-rich regions that are under a high selective pressure. Moreover, the identification of the avrRpv3.1 locus with a 30Kbp deletion involved in the interaction with the host Rpv3.1 loci also highlighted that within this deletion there are two closely-related genes that encode proteins with LWY-fold structural modules repeats [14]. LWY-folds are common features of avirulence genes such as the RxLR effectors composed by C-terminal motifs (W,Y and L) and repeated up to eight times, These genes are in rapidly evolving parts of the genome and under high selective pressure [66]. RXLR effectors present this LWY domain and the discrepancy found between the number of RxLR common to different *P. viticola* isolates may reflect pathogen adaptation and evolution.

### NLP and non-RxLR *P. viticola* effectors

Another class of effectors in oomycetes are the “necrosis- and ethylene inducing peptide 1 (NEP1)-like proteins” (NLPs). This class is highly conserved and can be divided into two groups: non-cytotoxic NLPs and those that can strongly induce necrosis [67, 68]. The NLPs that induce necrosis are only able to do so on dicot plants but not on monocots [69]. This class of pathogenic effectors is characterised by a conserved central region of the protein, a GHRHDWE motif, and two highly conserved cysteine residues in the N-terminal region [68]. NLPs have been identified in several genera, such as *Phytophthora, Pythium* and *Hyaloperonospora*, and have been described to trigger plant defence responses and cell death [70–72].

In *P. viticola*, eight NLP coding genes were identified in the genomes of the isolates INRA-PV221, JL-7-2, and PvitFEM01 [73], and 10 were described for the ZJ-1-1 isolate [74]. Only NLP7 was shown to be able to trigger cell death and enhance plant resistance to *P. capsici* when expressed in *N. benthamiana* and to *Hyaloperonospora arabidopsidis* when overexpressed in *A. thaliana* [73, 74]. NLP4, 5, and 10 have also been described to enhance *A. thaliana* resistance to *H. arabidopsidis* [74]. These newly identified immunity-inducing NLPs will also contribute to unravel grapevine defense genes that are currently unknown.

Non-RxLR effectors containing LWY domains have also been described. Pvit47 was identified as a WY/LWY domain containing candidate effector protein that present cell death-inducing activity in Nicotiana species but not in grapevine [47]. This effector is expressed in sporangia, germinated spores and during grapevine infection in isolate Pv221 collected in Blanquefort (France) [75]. Pvit33 presents only the WY domain, is also expressed in different stages of *P. viticola* infection and was shown to induce cell death in Nicotiana and grapevine [75] trough SGT1 signalling pathway (SGT1 is highly conserved component of some Skp1/Cullin/F-box protein (SCF)-type E3 ubiquitin ligase complexes, linked to HR induced responses in gene-for-gene systems [76].

### First in-planta *P. viticola* proteome reveals the presence of effectors in the host apoplast

The proteome of *P. viticola* was recently disclosed by untargeted proteomics of the host apoplast, from susceptible and tolerant grapevine leaves inoculated with the pathogen. The authors identified 164 proteins, 48 of which were predicted to be secreted. Two PvRxLRs (PVIT_0014142.T1 and PVIT_0015177.T1) were identified in the proteome during the interaction with the tolerant cultivar ‘Regent,’ that present the Rpv 3.1 background. Four CRN proteins (PVIT_0001451.T1, PVIT_0006190.T1, PVIT_0006424.T1 and PVIT_0025443.T1) were identified for both interactions [77, 78].

This study also described that during infection of the susceptible cultivar, *P. viticola* was able to disrupt the SA pathway and interfere with isoprenoid biosynthesis in the plant. These actions indicate that the pathogen is attempting to suppress plant defences to establish successful colonisation. In contrast, during the interaction with the tolerant cultivar, the presence of proteins, such as an ubiquinol terminal oxidase (PVIT_0010523.T1), suggests that *P. viticola* faces greater difficulty in differentiating infection structures. A *B. cinerea* mutant of this protein has shown defects in mycelial growth, sporulation, spore germination, and virulence [79]. It also attempts to manipulate the host’s metabolism and protect itself from the oxidative stress induced by the plant’s defense responses [77, 78].

### New performers in the interaction theatre: oomycete proteases and inhibitors

Studies have demonstrated the involvement of proteases in the regulation of plant immunity, making them promising targets for enhancing plant resistance. Various plant proteases play a role in activating pathogen-/microbe-triggered immunity (PTI/MTI), and proteases secreted by pathogens directly or indirectly affect host immunity components. Furthermore, both plant and pathogen proteases contribute to the establishment of effector-triggered susceptibility (ETS) or immunity (ETI). Proteases and their inhibitors are essential components of both plant and pathogen secretomes, which operate within the apoplast and facilitate close communication within the pathosystem [80–82] Inhibitors of proteolytic enzymes are regulatory proteins present in all the kingdoms. They are implicated in all processes that involve proteases to regulate their activity. Protease inhibitors can be classified into clans and families, according to their specificity towards the target protease [81, 83]. Two major families of protease inhibitors have been described in oomycetes, the Kazal-like serine protease inhibitors, and cystatin-like protease inhibitors. These have been extensively studied due to their involvement mainly on *Phytophthora*-host interactions. These protease inhibitors are ubiquitous in oomycetes, with many similarities between different *Phytophthora* spp. and *Plasmopara* spp. [44].

Kazal-type serine proteinase inhibitors are grouped into the family I1 protease inhibitors [83]. They are defined by a conserved amino acid sequence domain: six cysteine residues engaged in disulphide bonds arranged in a 1–5/2–4/3–6 pattern [84]. Kazal domains often occur in tandem arrays, and serine protease inhibitors can have one or several of these domains. Moreover, atypical Kazal domains with one to two disulphide bridges have also been described [85, 86]. The expression of these specific inhibitors has been associated with higher degrees of pathogen aggressiveness [87].

In *Phytophthora infestans*, the serine protease inhibitors PiEPI1 and PiEPI10 have been shown to interact and bind specifically to members of the subtilisin A class of serine proteases, which includes the tomato P69B subtilase [85, 86, 88]. The cystatin-like cysteine protease inhibitors PiEPIC1 and PiEPIC2B have been shown to interact with an apoplastic papain-like cysteine protease, inhibiting tomato PLCPs RCR3 and C14 [89, 90]. PiEPIC2B also binds to the *Phytophthora*-inhibited protease 1 (PIP1), consequently inhibiting tomato defence response [91].

Other examples of known protease inhibitors include PpEPI10, a homologue of PiEPI10 found in *P. palmivora*, which has been shown to inhibit HbSPA protease in the host plant *Hevea brasiliensis* [92, 93]. *Phytophthora mirabilis* PmEPIC1, an ortholog of PiEPIC1, inhibits the RCR3-like protease MRP2 in its host *Mirabilis jalapa* [94]. Transcriptome studies have shown that these inhibitors are mostly expressed early stages ofthe infection course [95]. Muthuswamy et al. observed that the expression of Kazal-like serine protease inhibitors from *Phytophthora capsici* occurs only in the first hours of infection [87].

Several protease inhibitors have also been identified in the genomes of various species in the Peronosporaceae family, with a variable number of putative genes coding for serine or cysteine protease inhibitors. In the *Pseudoperonospora humuli* apoplastic secretome, 32 enzyme inhibitors have been identified, of which five are protease inhibitors belonging to the Kazal-like serine protease inhibitor family [96]. In the *Plasmopara halstedii* genome, downy mildew of sunflower, 54 cysteine, 62 serine, and 15 aspartic protease genes were identified. Moreover, 23 protease inhibitors were identified, of which 19 were putative Kazal-like serine protease inhibitors and four putative cystatin-like cysteine protease inhibitors [97]. In *Peronospora tabacina*, the causal agent of downy mildew in *Nicotiana tabacum*, aspartyl, cysteine, and serine proteases were found to be secreted, and six Kazal-like serine protease inhibitors were detected [98]. In the causal agent of basil downy mildew, *Peronospora belbahrii*, 26 serine and cysteine proteases were identified, and only two protease inhibitor genes were found to be secreted, with sequence similarity to Kazal-like serine protease inhibitors [99]. To date, serine protease inhibitors have not been characterized in *P. viticola*, representing a gap in knowledge in what constitutes a promising and strong weapon in the molecular arsenal of plant pathogens.

The availability of the *P. viticola* genome and transcriptome is crucial to start to unravel which proteases and protease inhibitors are present during *P. viticola* infection. Research on different groups of effectors has shown that both the number of effectors and their sequence conservation can vary among the isolates of *P. viticola*, indicating that its secretome can be more or less specific towards the variety or cultivar of *Vitis vinifera*. As such, the secretome of one isolate can contain protease inhibitors specific to the proteases of one *Vitis vinifera* variety, which may render this variety more susceptible [100]. In addition, plant proteases can undergo structural modifications between tolerant and susceptible cultivars, which halts the ability of the pathogen inhibitor to identify and inhibit protease activity.

The identification of the interacting partners of these protease and protease inhibitors in grapevine-downy mildew interactions will elucidate the importance of these molecules in plant defence and in the mechanisms of pathogen infection. In 2012, Gindro et al. showed for the first time that commercial protease inhibitors of serine and cysteine proteases were able to diminish plant defense mechanisms against *P. viticola* infection [100]. In the same year, the construction of a cDNA library from *P. viticola* allowed for the analysis of its transcripts in its interaction with *Vitis vinifera* cv. ‘Muscat Ottonel,’ through expressed sequence tags (ESTs) [101]. The authors identified cysteine protease, cysteine protease inhibitors and Kazal-like serine protease inhibitors among more than 500 *P. viticola* genes [101].

## Conclusion

This review provides valuable insights into the genetic diversity, population structure, and adaptation strategies of *Plasmopara viticola*, the ethiological agent of grapevine downy mildew. The genetic diversity analysis made so far reveals distinct patterns among populations in different geographic regions, highlighting the influence of historical introductions and local environmental conditions. The emergence of five formae speciales highlights the pathogen’s ability to adapt to specific host environments and, thus, the relevance of this pathogen when planning agricultural practices. Apart from that, the breakdown of grapevine resistance genes provides critical insights into the ongoing challenges in sustainable viticulture, particularly in the context of evolving *P. viticola* populations capable of overcoming host resistance.

The study of effector molecules, such as RXLRs and CRNs, sheds light on the sophisticated infection strategies employed by *P. viticola*. The identification of diverse effectors, their role in manipulating host defense mechanisms, and the ongoing arms race between the pathogen and grapevine resistance genes contribute to our understanding of the molecular interactions underlying the disease. Additionally, the role of proteases and protease inhibitors in the *P. viticola*—grapevine interaction remains to be fully explored, which may provide a novel perspective on the pathogen’s ability to modulate host immunity.

In summary, this comprehensive review enhances our knowledge of *P. viticola* biology, epidemiology, and molecular mechanisms, offering valuable information for the development of effective disease management strategies in viticulture. It contributes to a broader understanding of oomycete-plant interactions and emphasize the need for continuous research to address emerging challenges in the context of evolving pathogen populations and changing environmental conditions.

### Supplementary Information

**Supplementary Material 1.**

## Data Availability

All data generated or analysed during this study are included in this published article [and its supplementary information files].

## Abbreviations

Avr: Avirulence factors
BAX: Bcl-2-associated X protein
BR: Brassinosteroid signaling
CAA: Carboxylic acid amide
CRN: Crinkler
ER: ERECTA signaling
ESTs: Expressed sequence tags
ET: Ethylene
ETI: Effector triggered immunity
ETS: Effector-triggered susceptibility
JA: Jasmonic acid
MAMPs: Microbe-associated molecular patterns
NEP1: Necrosis- and ethylene inducing peptide 1
NLPs: Necrosis- and ethylene inducing peptide 1-like proteins
NLR: Nod like receptor protein
NW: Neustadt ader Weinstrasse
PAMPs: Pathogen-associated molecular patterns
PTI/MPI: Pathogen-/microbe-trigerred immunity
R genes: Plant resistance genes
ROS: Reactive oxygen species
RPV: Resistance loci associated with *P. viticola*
SA: Salycilic acid
VIVC: Vitis International Variety Catalogue

## References

[1] Latijnhouwers M, de Wit PJGM, Govers F (2003). Oomycetes and fungi: similar weaponry to attack plants. Trends Microbiol.

[2] Thines M, Kamoun S (2010). Oomycete–plant coevolution: recent advances and future prospects. Curr Opin Plant Biol.

[3] Koledenkova K, Esmaeel Q, Jacquard C, Nowak J, Clément C, Ait BE (2022). *Plasmopara viticola* the causal agent of downy mildew of grapevine: from its taxonomy to disease management. Front Microbiol.

[4] McCarthy CGP, Fitzpatrick DA (2017). Phylogenomic reconstruction of the oomycete phylogeny derived from 37 genomes. mSphere.

[5] McGowan J, Fitzpatrick DA (2020). Chapter five - recent advances in oomycete genomics. Adv Genet.

[6] OIV - International Organisation of Vine and Wine (2023). State of the world vine and wine sector in 2022.

[7] Gessler C, Pertot I, Perazzolli M (2011). *Plasmopara viticola*: a review of knowledge on downy mildew of grapevine and effective disease management. Phytopathol Mediterr.

[8] Dussert Y, Mazet ID, Couture C, Gouzy J, Piron M-C, Kuchly C (2019). A high-quality grapevine downy mildew genome assembly reveals rapidly evolving and lineage-specific putative host adaptation genes. Genome Biol Evol.

[9] Dussert Y, Gouzy J, Richart-Cervera S, Mazet ID, Delière L, Couture C (2016). Draft genome sequence of *Plasmopara viticola*, the grapevine downy mildew pathogen. Genome Announc.

[10] Yin X, Liu RQ, Su H, Su L, Guo YR, Wang ZJ (2017). Pathogen development and host responses to *Plasmopara viticola* in resistant and susceptible grapevines: an ultrastructural study. Hortic Res.

[11] Armijo G, Schlechter R, Agurto M, Muñoz D, Nuñez C, Arce-Johnson P (2016). Grapevine pathogenic microorganisms: understanding infection strategies and host response scenarios. Front Plant Sci.

[12] Burruano S (2000). The life-cycle of *Plasmopara viticola*, cause of downy mildew of vine. Mycologist.

[13] Rossi V, Caffi T, Gobbin D (2013). Contribution of molecular studies to botanical epidemiology and disease modelling: grapevine downy mildew as a case-study. Eur J Plant Pathol.

[14] Paineau M, Minio A, Mestre P, Fabre F, Mazet ID, Couture C, et al. An effector deletion leads to the breakdown of partial grapevine resistance to downy mildew. bioRxiv. 2023;2023.08.17.553663. 10.1111/nph.19861 39021210

[15] Rouxel M, Mestre P, Comont G, Lehman BL, Schilder A, Delmotte F (2013). Phylogenetic and experimental evidence for host-specialized cryptic species in a biotrophic oomycete. New Phytol.

[16] Cadle-Davidson L (2008). Variation within and between *Vitis* spp. for foliar resistance to the downy mildew pathogen *Plasmopara viticola*. Plant Dis.

[17] Rouxel M, Mestre P, Baudoin A, Carisse O, Delière L, Ellis MA (2014). Geographic distribution of cryptic species of *Plasmopara viticola* causing downy mildew on wild and cultivated grape in Eastern North America. Phytopathology.

[18] Fontaine MC, Labbé F, Dussert Y, Delière L, Richart-Cervera S, Giraud T (2021). Europe as a bridgehead in the worldwide invasion history of grapevine downy mildew, *Plasmopara viticola*. Curr Biol.

[19] Schröder S, Telle S, Nick P, Thines M (2011). Cryptic diversity of *Plasmopara viticola* (Oomycota, Peronosporaceae) in North America. Org Divers Evol.

[20] Kump I, Blaise P, Gessler C (2000). The use of RAPD-markers to estimate genetic diversity of *Plasmopara viticola* in a single vineyard. Proceedings of the Third International Workshop on Grapevine Downy and Powdery Mildew.

[21] Stark-Urnau M, Seidel M, Kast WK, Gemmrich AR (2015). Studies on the genetic diversity of primary and secondary infections of *Plasmopara viticola* using RAPD/PCR. Vitis J Grapevine Res.

[22] Gobbin D, Pertot I, Gessler C (2003). Identification of microsatellite markers for *Plasmopara viticola* and establishment of high throughput method for SSR analysis. Eur J Plant Pathol.

[23] Gobbin D, Pertot I, Gessler C (2003). Genetic structure of a *Plasmopara viticola* population in an isolated Italian Mountain Vineyard. J Phytopathol.

[24] Gobbin D, Jermini M, Loskill B, Pertot I, Raynal M, Gessler C (2005). Importance of secondary inoculum of *Plasmopara viticola* to epidemics of grapevine downy mildew. Plant Pathol.

[25] Delmotte F, Chen WJ, Richard-Cervera S, Greif C, Papura D, Giresse X (2006). Microsatellite DNA markers for *Plasmopara viticola*, the causal agent of downy mildew of grapes. Mol Ecol Notes.

[26] Matasci CL, Jermini M, Gobbin D, Gessler C (2010). Microsatellite based population structure of *Plasmopara viticola* at single vine scale. Eur J Plant Pathol.

[27] Rouxel M, Papura D, Nogueira M, Machefer V, Dezette D, Richard-Cervera S (2012). Microsatellite markers for characterization of native and introduced populations of *Plasmopara viticola*, the causal agent of grapevine downy mildew. Appl Environ Microbiol.

[28] Fontaine MC, Austerlitz F, Giraud T, Labbé F, Papura D, Richard-Cervera S (2013). Genetic signature of a range expansion and leap-frog event after the recent invasion of Europe by the grapevine downy mildew pathogen *Plasmopara viticola*. Mol Ecol.

[29] Yin L, Zhang Y, Hao Y, Lu J (2014). Genetic diversity and population structure of *Plasmopara viticola* in China. Eur J Plant Pathol.

[30] Zhang W, Manawasinghe IS, Zhao W, Xu J, Brooks S, Zhao X (2017). Multiple gene genealogy reveals high genetic diversity and evidence for multiple origins of Chinese *Plasmopara viticola* population. Sci Rep.

[31] Koopman T, Linde CC, Fourie PH, McLeod A (2007). Population genetic structure of *Plasmopara viticola* in the Western Cape Province of South Africa. Mol Plant Pathol.

[32] Taylor AS, Knaus BJ, Grünwald NJ, Burgess T (2019). Population genetic structure and cryptic species of *Plasmopara viticola* in Australia. Phytopathology.

[33] Delmas CEL, Fabre F, Jolivet J, Mazet ID, Richart Cervera S, Delière L (2016). Adaptation of a plant pathogen to partial host resistance: selection for greater aggressiveness in grapevine downy mildew. Evol Appl.

[34] Peressotti E, Wiedemann-Merdinoglu S, Delmotte F, Bellin D, Di Gaspero G, Testolin R (2010). Breakdown of resistance to grapevine downy mildew upon limited deployment of a resistant variety. BMC Plant Biol.

[35] Heyman L, Höfle R, Kicherer A, Trapp O, Ait Barka E, Töpfer R (2021). The durability of quantitative host resistance and variability in pathogen virulence in the interaction between European grapevine cultivars and *Plasmopara viticola*. Front Agron.

[36] Gouveia C, Zukic S, Manthey T, Malhó R, Buchholz G, Figueiredo A (2021). Subtilisin like proteins in the war between Grapevine and *Plasmopara viticola* isolates with contrasting aggressiveness. Eur J Plant Pathol.

[37] Vezzulli S, Gramaje D, Tello J, Gambino G, Bettinelli P, Pirrello C, et al. Genomic designing for biotic stress resistant grapevine BT - genomic designing for biotic stress resistant fruit crops. In: Kole C, editor. Cham: Springer International Publishing; 2022. p. 87–255.

[38] Di Gaspero G, Copetti D, Coleman C, Castellarin SD, Eibach R, Kozma P (2012). Selective sweep at the Rpv3 locus during grapevine breeding for downy mildew resistance. Theor Appl Genet.

[39] Wingerter C, Eisenmann B, Weber P, Dry I, Bogs J (2021). Grapevine Rpv3-, Rpv10- and Rpv12-mediated defense responses against *Plasmopara viticola* and the impact of their deployment on fungicide use in viticulture. BMC Plant Biol.

[40] Paineau M, Mazet ID, Wiedemann-Merdinoglu S, Fabre F, Delmotte F (2022). The characterization of pathotypes in grapevine downy mildew provides insights into the breakdown of Rpv3, Rpv10, and Rpv12 factors in grapevines. Phytopathology.

[41] Fouché S, Badet T, Oggenfuss U, Plissonneau C, Francisco CS, Croll D (2020). Stress-driven transposable element de-repression dynamics and virulence evolution in a fungal pathogen. Mol Biol Evol.

[42] Dvorak E, Mazet I, Couture C, Foulongne-Oriol M, Delmotte F. Crossing *Plasmopara viticola* strains in controlled conditions to uncover the genomic bases of downy mildew resistance breakdown in grapevine. BIO Web Conf. 2022;50:02002.

[43] Bozkurt TO, Kamoun S (2020). The plant–pathogen haustorial interface at a glance. J Cell Sci.

[44] Stassen JHM, Van den Ackerveken G (2011). How do oomycete effectors interfere with plant life?. Curr Opin Plant Biol.

[45] Bozkurt TO, Schornack S, Banfield MJ, Kamoun S (2012). Oomycetes, effectors, and all that jazz. Curr Opin Plant Biol.

[46] Birch PRJ, Armstrong M, Bos J, Boevink P, Gilroy EM, Taylor RM (2009). Towards understanding the virulence functions of RXLR effectors of the oomycete plant pathogen *Phytophthora infestans*. J Exp Bot.

[47] Combier M, Evangelisti E, Piron M-C, Schornack S, Mestre P (2022). Candidate effector proteins from the oomycetes *Plasmopara viticola* and *Phytophthora parasitica* share similar predicted structures and induce cell death in Nicotiana species. PLoS ONE.

[48] Jiang RHY, Tyler BM (2011). Mechanisms and evolution of virulence in oomycetes. Annu Rev Phytopathol.

[49] Bos JIB, Kanneganti T-D, Young C, Cakir C, Huitema E, Win J (2006). The C-terminal half of *Phytophthora infestans* RXLR effector AVR3a is sufficient to trigger R3a-mediated hypersensitivity and suppress INF1-induced cell death in *Nicotiana benthamiana*. Plant J.

[50] Chepsergon J, Motaung TE, Moleleki LN (2021). “Core” RxLR effectors in phytopathogenic oomycetes: a promising way to breeding for durable resistance in plants?. Virulence.

[51] Yin J, Gu B, Huang G, Tian Y, Quan J, Lindqvist-Kreuze H (2017). Conserved RXLR effector genes of *Phytophthora infestans* expressed at the early stage of potato infection are suppressive to host defense. Front Plant Sci.

[52] Lan X, Liu Y, Song S, Yin L, Xiang J, Qu J (2019). *Plasmopara viticola* effector PvRXLR131 suppresses plant immunity by targeting plant receptor-like kinase inhibitor BKI1. Mol Plant Pathol.

[53] Xiang J, Li X, Wu J, Yin L, Zhang Y, Lu J (2016). Studying the mechanism of *Plasmopara viticola* RxLR effectors on suppressing plant immunity. Front Microbiol.

[54] Xiang J, Li X, Yin L, Liu Y, Zhang Y, Qu J (2017). A candidate RxLR effector from *Plasmopara viticola* can elicit immune responses in *Nicotiana benthamiana*. BMC Plant Biol.

[55] Fu Q, Wang Y, Yang J, Jiao Y, Li W, Yang F (2023). *Plasmopara viticola* RxLR effector PvAvh77 triggers cell death and governs immunity responses in grapevine. J Exp Bot.

[56] Lei X, Lan X, Ye W, Liu Y, Song S, Lu J (2019). *Plasmopara viticola* effector PvRXLR159 suppresses immune responses in *Nicotiana benthamiana*. Plant Signal Behav.

[57] Liu J, Chen S, Ma T, Gao Y, Song S, Ye W (2021). *Plasmopara viticola* effector PvRXLR53 suppresses innate immunity in *Nicotiana benthamiana*. Plant Signal Behav.

[58] Torto TA, Li S, Styer A, Huitema E, Testa A, Gow NAR (2003). EST mining and functional expression assays identify extracellular effector proteins from the plant pathogen *Phytophthora*. Genome Res.

[59] Xiang G, Yin X, Niu W, Chen T, Liu R, Shang B (2021). Characterization of CRN-like genes from *Plasmopara viticola*: searching for the most virulent ones. Front Microbiol.

[60] Liu Y, Lan X, Song S, Yin L, Dry IB, Qu J (2018). In planta functional analysis and subcellular localization of the oomycete pathogen *Plasmopara viticola* candidate RXLR effector repertoire. Front Plant Sci.

[61] Yin L, Li X, Xiang J, Qu J, Zhang Y, Dry IB (2015). Characterization of the secretome of *Plasmopara viticola* by *de novo* transcriptome analysis. Physiol Mol Plant Pathol.

[62] Mestre P, Carrere S, Gouzy J, Piron M-C, Tourvieille de Labrouhe D, Vincourt P (2016). Comparative analysis of expressed CRN and RXLR effectors from two *Plasmopara* species causing grapevine and sunflower downy mildew. Plant Pathol.

[63] Yin L, An Y, Qu J, Li X, Zhang Y, Dry I (2017). Genome sequence of *Plasmopara viticola* and insight into the pathogenic mechanism. Sci Rep.

[64] Brilli M, Asquini E, Moser M, Bianchedi PL, Perazzolli M, Si-Ammour A (2018). A multi-omics study of the grapevine-downy mildew (*Plasmopara viticola*) pathosystem unveils a complex protein coding-A nd noncoding-based arms race during infection. Sci Rep.

[65] Chen T, Liu R, Dou M, Li M, Li M, Yin X (2020). Insight into function and subcellular localization of *Plasmopara viticola* Putative RxLR effectors. Front Microbiol.

[66] Jiang RHY, Tripathy S, Govers F, Tyler BM (2008). RXLR effector reservoir in two *Phytophthora* species is dominated by a single rapidly evolving superfamily with more than 700 members. PNAS.

[67] Qutob D, Kemmerling B, Brunner F, Küfner I, Engelhardt S, Gust AA (2006). Phytotoxicity and innate immune responses induced by Nep1-like proteins. Plant Cell.

[68] Seidl MF, Van den Ackerveken G (2019). Activity and phylogenetics of the broadly occurring family of microbial Nep1-like proteins. Annu Rev Phytopathol.

[69] Bailey B (1995). Purification of a protein from culture Filtrates of *Fusarium oxysporum* that induces ethylene and necrosis in leaves of *Erythroxylum coca*. Phytopathology.

[70] Fellbrich G, Romanski A, Varet A, Blume B, Brunner F, Engelhardt S (2002). NPP1, a *Phytophthora*-associated trigger of plant defense in parsley and Arabidopsis. Plant J.

[71] Gijzen M, Nürnberger T (2006). Nep1-like proteins from plant pathogens: recruitment and diversification of the NPP1 domain across taxa. Phytochemistry.

[72] Oome S, Raaymakers TM, Cabral A, Samwel S, Böhm H, Albert I (2014). Nep1-like proteins from three kingdoms of life act as a microbe-associated molecular pattern in Arabidopsis. PNAS.

[73] Schumacher S, Grosser K, Voegele RT, Kassemeyer H-H, Fuchs R (2020). Identification and characterization of Nep1-like proteins from the grapevine downy mildew pathogen *Plasmopara viticola*. Front Plant Sci.

[74] Xiang J, Cheng J, Wei L, Li M, Wu J (2022). Functional analysis of the Nep1-like proteins from *Plasmopara viticola*. Plant Signal Behav.

[75] Combier M, Evangelisti E, Piron M-C, Rengel D, Legrand L, Shenhav L (2019). A secreted WY-domain-containing protein present in European isolates of the oomycete *Plasmopara viticola* induces cell death in grapevine and tobacco species. PLoS ONE.

[76] El Oirdi M, Bouarab K (2007). Plant signalling components EDS1 and SGT1 enhance disease caused by the necrotrophic pathogen *Botrytis cinerea*. New Phytol.

[77] Figueiredo J, Santos RB, Guerra-Guimarães L, Leclercq CC, Renaut J, Malhó R (2022). An in-planta comparative study of *Plasmopara viticola* proteome reveals different infection strategies towards susceptible and Rpv3-mediated resistance hosts. Sci Rep.

[78] Figueiredo J, Santos RB, de Guerra Guimarães LC, Leclercq CC, Renaut J, Sousa L (2022). Deep into the apoplast: grapevine and *Plasmopara viticola* proteomes reveal the secret beneath host and pathogen communication at 6h after contact. Phytopathology.

[79] Lin Z, Wu J, Jamieson PA, Zhang C (2019). Alternative oxidase is involved in the pathogenicity, development, and oxygen stress response of *Botrytis cinerea*. Phytopathology.

[80] Thomas EL, van der Hoorn RALL (2018). Ten prominent host proteases in plant-pathogen interactions. Int J Mol Sci.

[81] Jashni MK, Mehrabi R, Collemare J, Mesarich CH, de Wit PJGM (2015). The battle in the apoplast: further insights into the roles of proteases and their inhibitors in plant–pathogen interactions. Front Plant Sci.

[82] Santos RB, Figueiredo A (2021). Two sides of the same story in grapevine–pathogen interactions. J Exp Bot.

[83] Rawlings ND, Barrett AJ, Thomas PD, Huang X, Bateman A, Finn RD (2018). The MEROPS database of proteolytic enzymes, their substrates and inhibitors in 2017 and a comparison with peptidases in the PANTHER database. Nucleic Acids Res.

[84] Mistry J, Chuguransky S, Williams L, Qureshi M, Salazar GA, Sonnhammer ELL (2021). Pfam: the protein families database in 2021. Nucleic Acids Res.

[85] Tian M, Kamoun S (2005). A two disulfide bridge Kazal domain from *Phytophthora* exhibits stable inhibitory activity against serine proteases of the subtilisin family. BMC Biochem.

[86] Tian M, Benedetti B, Kamoun S (2005). A second Kazal-like protease inhibitor from *Phytophthora infestans* inhibits and interacts with the apoplastic pathogenesis-related protease P69B of tomato. Plant Physiol.

[87] Muthuswamy A, KakkattilBalakrishnan V, Palaniyandi U, Chandran MV (2018). Pathogenic variability in *Phytophthora capsici* from black pepper (*Piper nigrum* L.) as revealed by transcriptome analysis. Indian Phytopathol.

[88] Tian M, Huitema E, Da Cunha L, Torto-Alalibo T, Kamoun S (2004). A Kazal-like extracellular serine protease inhibitor from *Phytophthora infestans* targets the tomato pathogenesis-related protease P69B. J Biol Chem.

[89] Song J, Win J, Tian M, Schornack S, Kaschani F, Ilyas M (2009). Apoplastic effectors secreted by two unrelated eukaryotic plant pathogens target the tomato defense protease Rcr3. PNAS.

[90] Kaschani F, Van der Hoorn RAL (2011). A model of the C14-EPIC complex indicates hotspots for a protease-inhibitor arms race in the oomycete-potato interaction. Plant Signal Behav.

[91] Tian M, Win J, Song J, van der Hoorn R, van der Knaap E, Kamoun S (2007). A *Phytophthora infestans* cystatin-like protein targets a novel tomato papain-like apoplastic protease. Plant Physiol.

[92] Ekchaweng K, Evangelisti E, Schornack S, Tian M, Churngchow N (2017). The plant defense and pathogen counterdefense mediated by *Hevea brasiliensis* serine protease HbSPA and *Phytophthora palmivora* extracellular protease inhibitor PpEPI10. PLoS ONE.

[93] Chinnapun D, Tian M, Day B, Churngchow N (2009). Inhibition of a *Hevea brasiliensis* protease by a Kazal-like serine protease inhibitor from *Phytophthora palmivora*. Physiol Mol Plant Pathol.

[94] Dong S, Stam R, Cano LM, Song J, Sklenar J, Yoshida K (2014). Effector specialization in a lineage of the Irish potato famine pathogen. Science.

[95] Nellist CF, Armitage AD, Bates HJ, Sobczyk MK, Luberti M, Lewis LA (2021). Comparative analysis of host-associated variation in *Phytophthora cactorum*. Front Microbiol.

[96] Purayannur S, Gent DH, Miles TD, Radišek S, Quesada-Ocampo LM (2021). The hop downy mildew pathogen *Pseudoperonospora humuli*. Mol Plant Pathol.

[97] Sharma R, Xia X, Cano LM, Evangelisti E, Kemen E, Judelson H (2015). Genome analyses of the sunflower pathogen *Plasmopara halstedii* provide insights into effector evolution in downy mildews and *Phytophthora*. BMC Genomics.

[98] Derevnina L, Chin-Wo-Reyes S, Martin F, Wood K, Froenicke L, Spring O (2015). Genome sequence and architecture of the tobacco downy mildew pathogen *Peronospora tabacina*. Mol Plant-Microbe Interact.

[99] Thines M, Sharma R, Rodenburg SYA, Gogleva A, Judelson HS, Xia X (2020). The genome of *Peronospora belbahrii* reveals high heterozygosity, a low number of canonical effectors, and TC-Rich promoters. Mol Plant-Microbe Interact.

[100] Gindro K, Berger V, Godard S, Voinesco F, Schnee S, Viret O (2012). Protease inhibitors decrease the resistance of *Vitaceae* to *Plasmopara viticola*. Plant Physiol Biochem.

[101] Mestre P, Piron MC, Merdinoglu D (2012). Identification of effector genes from the phytopathogenic *Oomycete Plasmopara viticola* through the analysis of gene expression in germinated zoospores. Fungal Biol.

