# Supplementary material for: The pathogenicity of Plasmopara viticola: a review of evolutionary dynamics, infection strategies and effector molecules
Source: BMC Plant Biol. 2024 Apr 24;24:327. doi: 10.1186/s12870-024-05037-0 (PMC11040782; doi:10.1186/s12870-024-05037-0)
Supplement: Supplementary file 1 — Supplementary Material 1. [file 12870_2024_5037_MOESM1_ESM.docx]

Supplementary Table 1. *Plasmopara viticola* effectors described to date.

| **Strain** | **Effector protein** | **Gene name** | **Type of effector molecule** | **Necrosis and HR test** | **Gene expression** | **Characteristics, localization and possible protein function** | **References** |
| --- | --- | --- | --- | --- | --- | --- | --- |
| ZJ-1-1 | Secreted RxLR effector protein 1; 5; 22; 30 | *RxLR1; RxLR5; RxLR22; RxLR30* | RXLR | Suppresses cell death triggered by INF1 | Expression is up-regulated at the earlier infection stages. | Targeted to the host nucleus | [52] |
| ZJ-1-1 | Secreted RxLR effector protein 2; 9; 11; 17; 49; 68 | *RxLR2; RxLR9; RxLR11; RxLR17; RxLR49; RxLR68* | RXLR | Suppresses cell death triggered by INF1 | Expression is up-regulated at later stages of infection | Targeted to the host nucleus and cytoplasm | [52] |
| JL-7-2 | Secreted RxLR effector protein 3; 6; 7; 15; 21; 37;38; 40; 43; 48; 50; 57; 83; 89; 94; 135; 144; 146; 147; 149; 159 | *RXLR3; RXLR6; RXLR7; RXLR15; RXLR21; RXLR37; RXLR38; RXLR40; RXLR43; RXLR48; RXLR50; RXLR57; RXLR83; RXLR89; RXLR94; RXLR135; RXLR144; RXLR146; RXLR147; RXLR149; RXLR159* | RXLR | Completely suppresses the host cell death | - | Targeted to the host nucleus and cytoplasm | [47] |
| ZJ-1-1 | Secreted RxLR effector protein 10; 27 | *RXLR10; RXLR27* | RXLR | suppresses cell death triggered by Inf1 | Expression is up-regulated at the earlier infection stages. | Targeted to the host nucleus and cytoplasm | [52] |
| JL-7-2 | Secreted RxLR effector protein 8; 14; 36;39; 51; 69; 70; 91; 93; 104; 108; 118; 120; 134; 142; 152 | *RXLR8; RXLR14; RXLR36; RXLR39; RXLR51; RXLR69; RXLR70; RXLR91; RXLR93; RXLR104; RXLR108; RXLR118; RXLR120; RXLR134; RXLR142; RXLR152* | RXLR | Completely suppresses the host cell death | - | Targeted to the host nucleus | [47] |
| ZJ-1-1 | Secreted RxLR effector protein 16 | *RxLR16* | RXLR | Triggers cell death dependent on SGT1, HSP90 and RAR1 (Key proteins in regulating the stability of R protein complex) | Expression is up-regulated at the earlier infection stages. | Targeted to the host nucleus. Overexpression in *A. thaliana* enhances the expression of defense-associated genes involved in the salicylic acid-, jasmonate acid-, and ethylene-mediated signal transduction, promoting ROS accumulation and disease resistance | [52]  [53] |
|  | Secreted RxLR effector protein 18 | *RXLR18* |  | suppresses cell death | spores have strong up-regulation, and is up- regulated at earlier stages of infection | - | [29]  [81]  [47]  [48] |
| ZJ-1-1 | Secreted RxLR effector protein 19 | *RxLR19* | RXLR | partially suppresses the host cell death | Expression is up-regulated at later stages of infection | Targeted to the host nucleus and cytoplasm | [52] |
| JL-7-2 | Secreted RxLR effector protein 20; 81; 124; 150; 158 | *RXLR20; RXLR81; RXLR124; RXLR150; RXLR158* | RXLR | partially suppresses the host cell death | - | Targeted to the host nucleus and cytoplasm | [47] |
| JL-7-2 | Secreted RxLR effector protein 24; 82; 102; 111; 122; 138 | *RXLR24; RXLR82; RXLR102; RXLR111; RXLR122; RXLR138* | RXLR | Triggers cell death | - | Targeted to the Host nucleus | [47] |
| ZJ-1-1 | Secreted RxLR effector protein 25 | *RxLR25* | RXLR | partially suppresses programmed cell death | Expression is up-regulated at the earlier infection stages. | Targeted to the host nucleus and cytoplasm | [52] |
| ZJ-1-1 | Secreted RxLR effector protein 28 | *RxLR28* | RXLR | - | expression peaks at 6 hpi | PvRxLR28 Expression Enhances Plant Susceptibility to P. viticola and P. parasitica when transiently expressed in the leaves of grapevine and tobacco. by repressing the expression of the defense-related genes in plants as ROS-producing proteins | [52] |
| ZJ-1-1 | Secreted RxLR effector protein 29 | *RxLR29* | RXLR | Suppresses cell death triggered by INF1 | - | Targeted to the host nucleus and cytoplasm | [52] |
| JL-7-2 | Secreted RxLR effector protein 31; 41; 105; 115; 123 | *RXLR31; RXLR41; RXLR105; RXLR115; RXLR123* | RXLR | does not interfere | - | Targeted to the host nucleus and cytoplasm | [47] |
| JL-7-2 | Secreted RxLR effector protein 35 | *RXLR35* | RXLR | Triggers cell death | - | Targeted to the host nucleus | [47] |
| JL-7-2 | Secreted RxLR effector protein 41 | *RXLR41* | RXLR | does not interfere | - | Targeted to the host nucleus and cytoplasm | [47] |
| JL-7-2 | Secreted RxLR effector protein 45 | *RXLR45* | RXLR | completely suppresses the host cell death | - | Targeted to the host nucleus but not the nucleolus | [47] |
| JL-7-2 | Secreted RxLR effector protein 47; 126 | *RXLR47; RXLR 126* | RXLR | completely suppresses the host cell death | - | Targeted to the host cell membrane | [47] |
| JL-7-2 | Secreted RxLR effector protein 54 | *RXLR54* | RXLR | completely suppresses the host cell death | - | Targeted to the Chloroplast nuclei, and mitochondria | [47] |
| ZJ-1-1 | Secreted RxLR effector protein 55 | *RxLR55* | RXLR | suppresses cell death triggered by Inf1 | Expression is up-regulated at later stages of infection | Targeted to the host cell membrane | [52] |
| JL-7-2 and ZJ-1-1 | Secreted RxLR effector protein 61 | *RxLR61* | RXLR | partially suppresses programmed cell death | Expression is up-regulated at the earlier infection stages. | Targeted to the Chloroplast nuclei, cytoplasm and nucleus | [52]  [47] |
| JL-7-2 | Secreted RxLR effector protein 63; 76;101 | *RxLR63; RxLR76; RxLR101* | RXLR | partially suppresses programmed cell death | - | Targeted to the host nucleus | [47] |
| ZJ-1-1 | Secreted RxLR effector protein 64 | *RxLR64* | RXLR | suppresses cell death triggered by Inf1 | Expression is up-regulated at the earlier infection stages | Targeted to the host nucleus, cytoplasm and cell membrane | [52] |
| ZJ-1-1 | Secreted RxLR effector protein 66 | *RxLR66* | RXLR | Inhibits cell death | Expression is up-regulated at later stages of infection | Targeted to the host nucleus, cytoplasm and cell membrane | [52 |
| ZJ-1-1 | Secreted RxLR effector protein 67 | *RxLR67* | RXLR | partially suppresses programmed cell death | Expression is up-regulated at later stages of infection | Targeted to the host nucleus, cytoplasm and cell membrane | [52] |
|  | avirulence homolog (Avh) RxLR effector 77 | *PvAvh77* | RXLR | Triggers cell death in *N. benthamiana* and in *V. riparia* | strongly up-regulated during the initial stages of *P. viticola* infection | Targeted to the nucleus, enhances colonization by P. viticola in V. vinifera ‘Thompson Seedless’ leaves when overexpressed. But application of purified protein in leaves induces plant immunity. | [54] |
| JL-7-2 | Secreted RxLR effector protein 80 | *RXLR80* | RXLR | does not interfere | - | Targeted to the endoplasmatic reticulum | [47] |
| JL-7-2 | Secreted RxLR effector protein 81 | *RXLR81* | RXLR | partially suppresses the host cell death | - | Targeted to the host nucleus and cytoplasm | [47] |
| JL-7-2 | Secreted RxLR effector protein 85 | *RXLR85* | RXLR | partially suppresses the host cell death | - | Targeted to the plasma membrane | [47] |
| JL-7-2 | Secreted RxLR effector protein 86 | *RXLR86* | RXLR | does not interfere | - | Targeted to the chloroplast, has a N-terminal cleavable transit peptide | [47] |
| JL-7-2 | Secreted RxLR effector protein 90; 143; 153; 154; 160 | *RXLR90; RXLR143; RXLR153; RXLR154; RXLR160* | RXLR | completely suppresses the host cell death | - | Targeted to the plasma membrane | [47] |
| JL-7-2 | Secreted RxLR effector protein 95 | *RXLR95* | RXLR | induces cell death | - | Targeted to the host nucleus and cytoplasm | [47] |
| JL-7-2 | Secreted RxLR effector protein 100; 128 | *RXLR100; RXLR128* | RXLR | does not interfere | - | Targeted to the host nucleus | [47] |
| JL-7-2 | Secreted RxLR effector protein 105 | *RXLR105* | RXLR | does not interfere | - | Targeted to the host nucleus and cytoplasm | [47] |
| JL-7-2 | Secreted RxLR effector protein 115 | *RXLR115* | RXLR | does not interfere | - | Targeted to the host nucleus and cytoplasm | [47] |
| JL-7-2 | Secreted RxLR effector protein 117 | *RXLR117* | RXLR | induces cell death | - | Targeted to the host nucleus | [47] |
| JL-7-2 | Secreted RxLR effector protein 123 | *RXLR123* | RXLR | does not interfere | - | Targeted to the host nucleus and cytoplasm | [47] |
| JL-7-2 | Secreted RxLR effector protein 124 | *RXLR124* | RXLR | partially suppresses the host cell death | - | Targeted to the host nucleus and cytoplasm | [47] |
| JL-7-2 | Secreted RxLR effector protein 131 | *RXLR131* | RXLR | suppresses the host cell death | Expression is induced during infection of host grapevine, peaking at 36 hpi | targeted ton the host membrane, interacts with host BRI1 kinase inhibitor 1 (BKI1), consequently suppresses the brassinosteroid (BR) and ERECTA (ER) signaling pathways in planta, both related to the defense mechanism. | [51] |
| JL-7-2 | Secreted RxLR effector protein 150 | *RXLR150* | RXLR | partially suppresses the host cell death | - | Targeted to the host nucleus and cytoplasm | [47] |
| JL-7-2 | Secreted RxLR effector protein 151 | *RXLR151* | RXLR | completely suppresses the host cell death | - | Targeted to the endoplasmatic reticulum | [4] |
| JL-7-2 | Secreted RxLR effector protein 158 | *RXLR158* | RXLR | partially suppresses the host cell death | - | Targeted to the host nucleus and cytoplasm | [47] |
| JL-7-2 | Secreted RxLR effector protein 161 | *RXLR161* | RXLR | completely suppresses the host cell death | - | Targeted to the Chloroplast nuclei, has a predicted RNase H-like domain in the C-terminal region | [47] |
| ZJ-1-1 | NLP effector protein 1 (Nep1-like protein 1) | *NLP1* | necrosis inducing protein | does not induces cell death in Vitis leaves or in N. benthamiana | Highly expressed only in the early stages of infection, peaking with haustoria formation and decreasing during hyphae braching | Targeted to the host cytoplasm. NLP1 is assumed to be a pseudogene | [57] |
| ZJ-1-1 | NLP effector protein 2 (Nep1-like protein 2) | *NLP2* | necrosis inducing protein | does not induces cell death in Vitis leaves or in N. benthamiana | - | Targeted to the host cytoplasm, may act as a pathogen-associated molecular pattern (PAMP) | [57]  [58] |
| ZJ-1-1 | NLP effector protein 3 (Nep1-like protein 3) | *NLP3* | necrosis inducing protein | does not induces cell death in Vitis leaves or in N. benthamiana | Highly expressed only in the early stages of infection | Targeted to the host cytoplasm, may act as a pathogen-associated molecular pattern (PAMP) | [57]  [58] |
| ZJ-1-1 | NLP effector protein 4 (Nep1-like protein 4) | *NLP4* | necrosis inducing protein | does not induce necrosis in Nicotiana benthamiana leaves | Highly expressed only in the early stages of infection | improves disease resistance of Arabidopsis thaliana to Hyaloperonospora arabidopsidis when over-expressed in Arabidopsis | [58] |
| ZJ-1-1 | NLP effector protein 5 (Nep1-like protein 5) | *NLP5* | necrosis inducing protein | does not induce necrosis in Nicotiana benthamiana leaves | Highly expressed in the entire host infection process | improves disease resistance of Arabidopsis thaliana to Hyaloperonospora arabidopsidis when over-expressed in Arabidopsis | [58] |
| ZJ-1-1 | NLP effector protein 6 (Nep1-like protein 6) | *NLP6* | necrosis inducing protein | - | - | may act as a pathogen-associated molecular pattern (PAMP) | [57] |
| ZJ-1-1 | NLP effector protein 7 (Nep1-like protein 7) | *NLP7* | necrosis inducing protein | Triggers cell death | Highly expressed only in the early stages of infection | may act as a pathogen-associated molecular pattern (PAMP); can inducedisease resistance to Phytophthora capsici in Nicotiana benthamiana;improves disease resistance of Arabidopsis thaliana to Hyaloperonospora arabidopsidis | [57]  [58] |
| ZJ-1-1 | NLP effector protein 8 (Nep1-like protein 8) | *NLP8* | necrosis inducing protein | - | - | may act as a pathogen-associated molecular pattern (PAMP) | [57] |
| ZJ-1-1 | NLP effector protein 9 (Nep1-like protein 9) | *NLP9* | necrosis inducing protein | does not induce necrosis in Nicotiana benthamiana leaves | Highly expressed in the entire host infection process | - | [58] |
| ZJ-1-1 | NLP effector protein 10 (Nep1-like protein 10) | *NLP10* | necrosis inducing protein | does not induce necrosis in Nicotiana benthamiana leaves | Highly expressed in the entire host infection process | improves disease resistance of Arabidopsis thaliana to Hyaloperonospora arabidopsidis when over-expressed in Arabidopsis | [58] |
| YL | PvCRN1 | *PvCRN1* | CRN | does not interfere | - | Targeted to the host plasma membrane and nucleus, promotes resistance to P. capsici in N. benthamiana leaves | [40] |
| YL | PvCRN2 | *PvCRN2* | CRN | partially suppresses the host cell death triggered by INF1 and Bax in N. benthamiana | high expression at 96 hours post inculation | Targeted to the host plasma membrane and nucleus | [40] |
| YL | PvCRN4 | *PvCRN4* | CRN | does not interfere | - | Targeted to the host plasma membrane and nucleus | [40] |
| YL | PvCRN6 | *PvCRN6* | CRN | does not interfere | high expression at 72 and 96 hours post inculation | Targeted to the host plasma membrane and nucleus | [40] |
| YL | PvCRN7; 9; 21 | *PvCRN7; PvCRN9; PvCRN21* | CRN | does not interfere | high expression at 72 hours post inculation | Targeted to the host plasma membrane and nucleus | [40] |
| YL | PvCRN10 | *PvCRN10* | CRN | partially suppresses the host cell death triggered by Bax | high expression at 96 hours post inculation | Targeted to the host plasma membrane and nucleus, enhanced the resistance of N. benthamiana leaves to P. capsici | [40] |
| YL | PvCRN11 | *PvCRN11* | CRN | Triggers cell death in *N. benthamiana* but not *V. Vinifera* or *V. riparia* | - | Targeted to the host plasma membrane and nucleus, repressed the extension of P. capsici lesions on N. benthamiana leaves | [40] |
| YL | PvCRN12; 15; 22; 24 | *PvCRN12; PvCRN15; PvCRN22; PvCRN24* | CRN | partially suppresses the host cell death triggered by Bax | - | Targeted to the host plasma membrane and nucleus | [40] |
| YL | PvCRN14 | *PvCRN14* | CRN | partially suppresses the host cell death triggered by Bax | high expression at 72 and 96 hours post inculation | Targeted to the host plasma membrane and nucleus | [40] |
| YL | PvCRN16; 17 | *PvCRN16; PvCRN17* | CRN | partially suppresses the host cell death triggered by INF1 and Bax in *N. benthamiana* | high expression at 72 and 96 hours post inculation | Targeted to the host plasma membrane | [40] |
| YL | PvCRN18; 25 | *PvCRN18; PvCRN25* | CRN | partially suppresses the host cell death triggered by Bax | high expression at 96 hours post inculation | Targeted to the host plasma membrane and nucleus | [40] |
| YL | PvCRN19 | *PvCRN19* | CRN | does not interfere | - | Targeted to the host nucleus, significantly enhanced the susceptibility of N. benthamiana leaves to P. capsici | [40] |
| YL | PvCRN20 | *PvCRN20* | CRN | completely suppresses the host cell death triggered by INF1 and Bax in *N. benthamiana* | - | Targeted to the host plasma membrane and nucleus, promotes P. capsici colonization of N. benthamiana leaves | [40] |
| YL | PvCRN23 | *PvCRN23* | CRN | partially suppresses the host cell death triggered by Bax | - | Targeted to the host plasma membrane and nucleus, promotes P. capsici colonization of N. benthamiana leaves | [40] |
| YL | PvCRN26 | *PvCRN26* | CRN | partially suppresses the host cell death triggered by Bax | - | Targeted to the host plasma membrane and nucleus, enhanced the resistance of N. benthamiana leaves to P. capsici | [40] |
| YL | PvCRN27 | *PvCRN27* | CRN | does not interfere | - | Targeted to the host nucleus, improved the resistance level of N. benthamiana to the pathogen | [40] |
| YL | PvCRN29 | *PvCRN29* | CRN | does not interfere | high expression at 96 hours post inculation | Targeted to the host nucleus, improved the resistance level of N. benthamiana to the pathogen | [40] |
| YL | PvCRN30 | *PvCRN30* | CRN | partially suppresses the host cell death triggered by Bax | high expression at 96 hours post inculation | Targeted to the host plasma membrane | [40] |
| YL | PvCRN31 | *PvCRN31* | CRN | does not interfere | - | Targeted to the host plasma membrane and nucleus | [40] |
| YL | PvCRN35 | *PvCRN35* | CRN | partially suppresses the host cell death triggered by Bax | high expression at 96 hours post inculation | Targeted to the host plasma membrane and nuclear envelope | [40] |
